# Pattern of patients and diseases during mass transit: The day of Arafat experience

**DOI:** 10.12669/pjms.315.8017

**Published:** 2015

**Authors:** Abdulfattah I. Sindy, Mostafa Jamil Baljoon, Nadeem Alam Zubairi, Khalid Obaid Dhafar, Zohair Jamil Gazzaz, Basma Abdulhameed Deiab, FauzeaTalea Al Hothali

**Affiliations:** 1 Dr. Abdulfattah I. Sindy, MB ChB. Al-Noor Specialist Hospital, Makkah, Saudi Arabia; 2 Dr. Mostafa Jamil Baljoon, PhD. Qunfudah Health Affairs; 3 Dr. Nadeem Alam Zubairi, FCPS. Rabigh Medical College, King Abdulaziz University, Saudi Arabia; 4 Dr. Khalid Obaid Dhafar, FRCS. Al-Noor Specialist Hospital, Makkah, Saudi Arabia; 5 Dr. Zohair Jamil Gazzaz, PhD. Rabigh Medical College, King Abdulaziz University, Saudi Arabia; 6 Dr. Basma Abdulhameed Deiab, PhD. General Directorate of healthcare affairs, Makkah Region, Saudi Arabia; 7 FauzeaTalea Al Hothali, N.Dip. Makkah Region Nursing Administration, Saudi Arabia

**Keywords:** Hajj, Arafat, Saudi Arabia, flu vaccination

## Abstract

**Background and Objective:**

Every year 2-3 million Muslims gather for a few days around the Holy city of Makkah in Saudi Arabia to perform Hajj. Managing enormous health issues associated with such a mass gathering requires a very vibrant health delivery plan. Related research is part of the strategy. This study was done to assess the pattern of patients and illnesses encountered at one health facility at Arafat on the 2nd day of Hajj, when all the pilgrims move from Mina and stay in Arafat for a few hours. The objective of the study was to provide input so that recommendations can be given for future improvement of health care during this mass transit.

**Methods:**

All patients reporting sick to the Nimra Hospital on the Day of Arafat were included and documented on a detailed Performa and analyzed.

**Results:**

We received 211 patients, essentially all of those were in need of acute medical intervention. Acute severe asthma and injuries were the major problems encountered. There were two deaths both related to heat stroke. Patients received were predominantly Arabic speaking.

**Conclusions:**

Only those needing acute intervention seek medical advice during transit. Well equipped and staffed health facilities are, however, needed to cater these and for any mass casualties. Pre Hajj training and mandatory Flu vaccination can help.

## INTRODUCTION

Hajj is a yearly Islamic event during which millions of Muslim men and women gather in the surroundings of the Holy city of Makkah in Kingdom of Saudi Arabia (KSA). The actual pilgrimage is of 5 days duration but pilgrims, in particular those who have travelled from outside the Middle East, tend to stay for longer periods of time either before the start of Hajj or after the event.

Out of the 5 days of Hajj, pilgrims’ main stay is in the plains of Mina adjacent to Makkah, where they perform different rituals. However, on the second day there is a mass movement of all pilgrims from Mina to the plains of Arafat starting from dawn until afternoon. This is known as “The Day of Arafat”. All pilgrims then leave Arafat after sunset to spent night at Muzdalifa before going back to their tents in Mina.

The Ministry of Health (MOH) in KSA, in association with the Ministry of Hajj, caters for the health issues related to all pilgrims during the Hajj season. It also involves International collaboration for mutual benefits and to share the vast experience.^1^,^2^ Besides mobilizing the local resources, it temporarily employs highly qualified doctors, paramedics and related staff from other parts of the Muslim world. All levels of medical care to pilgrims is free of charge. MOH has well equipped and staffed hospitals and health facilities in Mina, with back up support in Makkah city to look after any expected or unexpected emergencies besides routine ailments. Hospitals and health centers have been established in Arafat to provide care to any medical emergencies during the short stay of pilgrims there while they are in transit.^3^,^4^ This study was undertaken to explore the characteristics of patients and their illnesses reporting sick in Arafat with the aim to subsequently use this information for allocation and adjustment of facilities there in coming Hajj seasons.

## METHODS

### Hajj 2013/1434H

Due to expansion projects underway in the Holy Mosque at Makkah, the quota for pilgrims from within the KSA and from outside Kingdom was markedly reduced. Still 1,980,249 registered pilgrims performed Hajj in 2013, 37 % less than the previous year.^5^ As explained earlier, all of these pilgrims did the mass movement from Mina to Arafat on the second day of Hajj before leaving for Muzdalifa on the same day after sun set. Nimra Hospital, where this study was done is one of the four hospitals situated in Arafat. It has a bed capacity of 90, with an ER and 15 clinics. Arafat has three other relatively bigger hospitals, namely Arafat General, Jabal-ur-Rahma and East Arafat hospitals with bed capacities of 300, 140 and 236 respectively. Nimra Hospital along with other three hospitals, being well equipped and staffed, rendered services throughout the Hajj season. However, the present study is concerned specifically to the Day of Arafat.

All patients reporting sick to the Nimra Hospital on the Day of Arafat were included and documented. A detailed Performa was required to be filled by the medical staff. This included details related to patient, his/her illness and disposal. The documents were collected by the Department of Quality and Patient Safety, MOH, Makkah Region and have been analyzed in the study using SPSS version 19.0.

## RESULTS

We received 211 patients in our facility during this transition. There were 136 males (64.4%) and 75 females (35.6%). Majority of the patients were between 50 to 69 years of age, but in general patients were evenly distributed for all age groups except for the extremes of ages. Fig.I. This was in accordance with the overall age pattern of pilgrims. The oldest patient was 88 years old while the youngest was an infant accompanying parents. Out of 211, we had 101 patients from Egypt, 34 from KSA and 18 from Pakistan besides pilgrims from many other countries. Fig-II.

**Fig.I:**
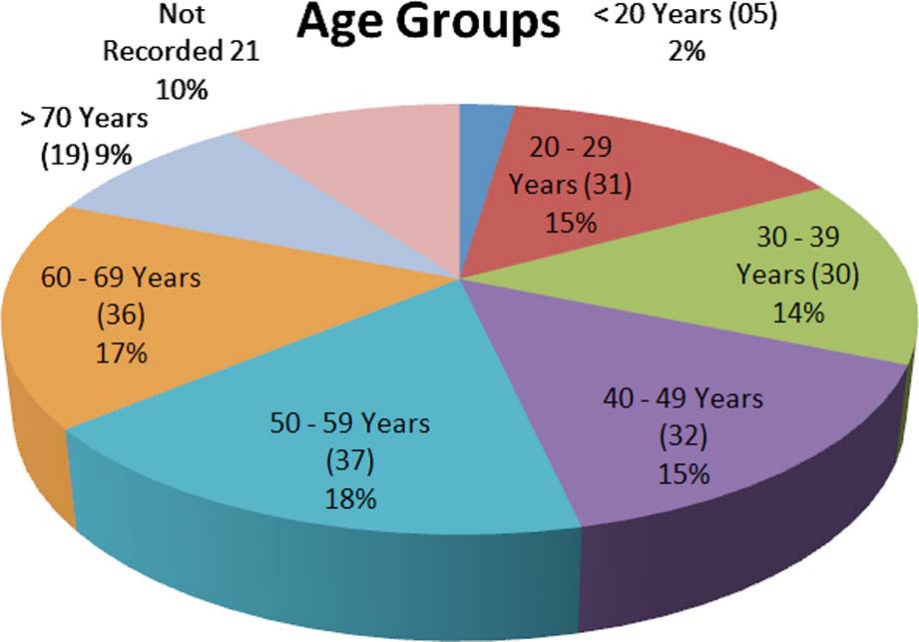
Age of Patients.

**Fig-II:**
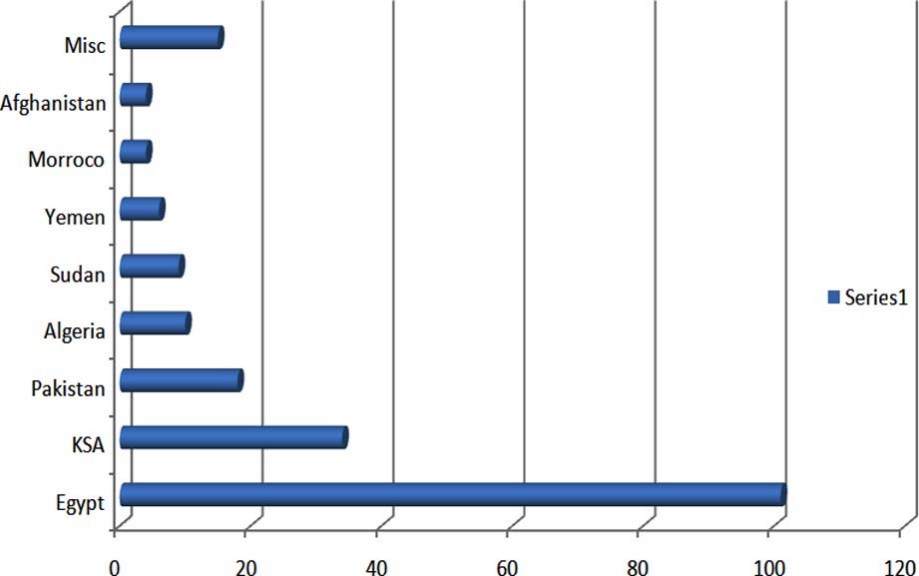
Patients Nationalities. KSA= Kingdom of Saudi Arabia.

### Disease pattern and outcome

Patients with Bronchial Asthma (34) and injuries (33) were the most common amongst a variety of conditions for which patients reported sick to our hospital in Arafat during this transitory phase. Out of 33 injuries, 8 were of a grave nature. Details of conditions encountered are in Table-I. Miscellaneous causes includes conditions seen only once like Epilepsy.

**Table-I:** Disease Pattern.

Bronchial Asthma	34
Injuries	33
Diabetes Mellitus	22
Associated DM	12
Heat effects	14
I.H.D	13
Arrhythmias	4
Hypertension	13
Associated HTN	3
Gastroenteritis	14
Dehydration	12
C.R.F	8
U.T.I	7
Renal colic	6
Confusional state	4
Acute Abdomen	7
C.V.A	3
Miscellaneous	17

Associated DM= Diabetes Mellitus with other major disease (not included in the count). Associated HTN= Hypertension with other major disease (not included in the count). IHD= Ischemic Heart Disease, CRF= Chronic Renal Failure, UTI= Urinary Tract Infection, CVA= Cerebro-Vascular Accident.

Out of 211 patients received at our Arafat medical facility on that day, there were two deaths. One patient was brought in dead with a history suggestive of heat stroke while the other, a 50 year old male, also had heat stroke, died while in hospital. Only six patients were referred/transferred to main hospitals in Mina and Makkah. They included 3 road accident patients and one each with peritonitis, cerebro-vascular-accident and chronic renal failure. Eleven patients were advised to stay but left against medical advice while remaining 192 were discharged in stable condition after receiving necessary treatment and care. Table-II.

**Table-II:** Outcome of Patients.

Outcome	Patients no.
Treated and Discharged	192
Referred to Tertiary Care Hospital	6
Deaths	2
Left Against Medical Advice	11

## DISCUSSION

Hajj offers a unique opportunity for people from all over the world to gather at one place for a few days. Bulk of the pilgrims are from different Islamic states besides local Saudi and non-Saudi residents but Muslims from essentially all parts of the world are there to perform the pilgrimage. There were persons from 188 countries during last Hajj with much variability in terms of ages, health status, educational level, etc.^6^ Hajj poses a great administrative challenge to concerned authorities. Logistics, transportation and health care are major areas of concern.^7^ The Saudi Arabian government works all the year round to ensure the smooth performance of Hajj, providing all available assistance and facilities to the pilgrims.^8^ Based on previous experience, these services remain in constant process of improvement. Research is encouraged.^9^ Center of Excellence in Hajj and Omrah Research (CEHOR) and Health at Hajj and Umrah (HAHU) Research groups are two examples. Much of the previous work in medical field has been targeting the aspects related to communicable diseases.^10^,^11^ It appears logical as this remains the biggest health threat to all pilgrims.

Studies done include evaluation and prevention of:

-Infection coming to Saudi Arabia during inflow of pilgrims.^12^,^13^
-Infection among pilgrims during the Hajj.^14^
-Infection getting exported to other countries with outgoing pilgrims.^15^

In our study, we concentrated on the health problems related to the 2nd day of Hajj when all pilgrims move to Arafat and usually stay there for a few hours before moving out.

Since the pilgrims are in transit and have to perform certain rituals in a stipulated time frame, the general tendency is not to seek medical advice for problems which can be deferred while they are in transition. This was apparent in our study, as 204 patients out of a total of 211 had problems where immediate intervention of variable levels was actually needed. This justifies the presence of advanced health care facilities in Arafat even for a day. Although 211 reporting sick out of around 2 million appears very small a percentage but the output of our medical facility to decrease both the mortality and morbidity among these 211 patients is considered worthwhile.

We had 34 patients with bronchial asthma needing temporary stay for nebulization and medication. Asthma is a common problem and most patients are carrying their inhalers and medications while coming for Hajj. Advanced symptoms not responding to their regular and ongoing medication need further medical help. Worsening of symptoms may be related to respiratory infections prevailing during this mass gathering. Hajj cough is a known entity.^16^ Influenza and Respiratory Syncytial Virus (RSV) have been identified as the main culprits.^17^ Unlike Meningococcal Vaccine which is mandatory, Influenza vaccine is not mandatory but recommended for all pilgrims.^18^,^19^ Individuals with better health awareness and those coming from countries like UK and USA tend to have this vaccination but this is not a common practice among pilgrims from developing world. Related to this, the threat of Middle East Respiratory Syndrome (MERS) provided a unique opportunity to put active detection, surveillance and international collaboration in place during the 2013 Hajj.^20^

Patients reporting with injuries were 33 in number. Eight of them had injuries of serious nature. Three had RTA (road traffic accident) needing referrals to Makkah after initial care and stabilization while 5 had fractures which were reduced and discharged from our facility. This emphasizes the importance of having a good surgical team with adequate facilities. Moreover, any incident of a larger scale with multiple casualties remains a possibility during such a mass gathering. Medical teams need to be well equipped in terms of staff and material for providing immediate triage and life saving measures before larger scale efforts are mobilized. Although no such event has occurred during last many years, the medical team had adequate resources with an effective contingency plan to manage such casualties.

Our facility received 22 patients with unstable diabetes mellitus (DM), while an additional 12 patients had DM but reported with other complaints. Since most of the pilgrims are in the age group where DM is common, cases were expected in Mina but to have numerous patients presenting with uncontrolled blood sugar levels while in transition at Arafat was unexpected. This indicates the need of endocrinologists not only at the Mina and Makkah hospitals but also at the Arafat centers.

Cases related to cardiac problems were expected. A gradual increase in admissions due to cardiovascular diseases has been noted in the last few years among the Hajj pilgrims.^21^ We encountered cases of Ischemic Heart Disease (IHD) and arrhythmias besides numerous patients having hypertension.

Heat effects with heat stroke and exhaustion remains a major threat during this transitory period. Efforts have been made at various levels to educate the pilgrims about the precautionary measures to be taken against heat effects. Plantation, shelters, showers and ample availability of fluids has aided in reducing such cases. Cooling facilities and heat stroke centers were established at all health facilities at Arafat. We only received 14 patients with heat effects. In addition, we also had 14 patients having gastroenteritis and 12 patients with dehydration without any symptom of gastroenteritis. Once again the reduction in number of gastroenteritis cases indicate successful pre-Hajj education of pilgrims by their respective countries and enormous improvement in sanitary and hygienic conditions during Hajj by the Saudi authorities.^22^ Gastroenteritis used to be one of the major causes of morbidity and mortality in the past, this is not the case anymore.^23^,^24^ Considering that the next 15 to 16 Hajj seasons will be in increasing hot summer months, the importance of proper education of pilgrims regarding prevention against heat effects cannot be over emphasized. Health teams have to be on guard as well.

Patients presenting with kidney related problems were 21 in number which included chronic renal failure, renal colic and urinary tract infection. There was no case of snake bite but one patient reported with scorpion bite. Four out of 75 female patients were pregnant though only one presented with threatened abortion.

The majority of patients, 176 in number, reporting sick were Arabic speaking (83.4%). This percentage is much higher than their ratio among all pilgrims and may indicate reluctance of non-Arabic speaking pilgrims to seek medical help due to fear of language barrier. This observation needs to be compared with statistics from Mina hospitals. Role of foreign medical missions is also there but these are mainly rendering outpatient services and are not handling emergencies in general. There were no patients from Indonesia and Malaysia, despite large number of pilgrims from these two countries. Once again their attendance in Mina health facilities needs to be compared. The location of their camps in Arafat relative to our hospital is one explanation.

In summary, although the number of pilgrims reporting sick to our facility at Arafat during the transitory period were not very large but essentially all were in need of acute medical intervention. The nature of illnesses encountered during the short period of pilgrims’ stay in Arafat indicates that the medical facilities there should have the services of cardiologists, endocrinologists, general specialists in internal medicine, orthopedic and general surgeons, general doctors, specialist nurses and a paramedical team.

### Limitations of the study

The study covers the patients attending just one facility at Arafat, hence does not give a comprehensive picture of the situation as there are many other hospitals an out patient clinics in the area. As such the results cannot be generalized.

## RECOMMENDATIONS

Arafat Hospitals should continue to have the services of adequate numbers of surgeons and specialists in internal medicine. Presence of cardiologists, endocrinologists and orthopedic surgeons is essential.

Considering high prevalence of respiratory infections during Hajj and their adverse effects on worsening the condition of patients with underlying respiratory pathologies like asthma, it is recommended that use of Influenza vaccine should be mandatory like meningococcal vaccine and not optional. Presence of large number of old aged pilgrims also supports this.

More emphasis is required on pre-Hajj education of pilgrims and more plantation and shelters at Arafat will be beneficial to counter an expected rise in cases of heat stroke and heat exhaustion in coming years when Hajj will be performed in hotter months.

Distribution of handouts in native languages regarding health tips and how to seek medical help will also help.
